# Recombinant Adiponectin Does Not Lower Plasma Glucose in Animal Models of Type 2 Diabetes

**DOI:** 10.1371/journal.pone.0044270

**Published:** 2012-10-01

**Authors:** Søren Tullin, Anette Sams, Jakob Brandt, Kirsten Dahl, Wei Gong, Claus Bekker Jeppesen, Thomas Nylandsted Krogh, Grith Skytte Olsen, Yun Liu, Anette Amstrup Pedersen, Jørn Meidahl Petersen, Bidda Rolin, Per-Olof Wahlund, Christoph Kalthoff

**Affiliations:** 1 Novo Nordisk A/S, Novo Nordisk Park, Måløv, Denmark; 2 Novo Nordisk R/D Center China, Changping District, Beijing, China; Virgen Macarena University Hospital, Spain

## Abstract

**Aims/Hypothesis:**

Several studies have shown that adiponectin can lower blood glucose in diabetic mice. The aim of this study was to establish an effective adiponectin production process and to evaluate the anti-diabetic potential of the different adiponectin forms in diabetic mice and sand rats.

**Methods:**

Human high molecular weight, mouse low molecular weight and mouse plus human globular adiponectin forms were expressed and purified from mammalian cells or yeast. The purified protein was administered at 10–30 mg/kg i.p. b.i.d. to diabetic db/db mice for 2 weeks. Furthermore, high molecular weight human and globular mouse adiponectin batches were administered at 5–15 mg/kg i.p. b.i.d. to diabetic sand rats for 12 days.

**Results:**

Surprisingly, none of our batches had any effect on blood glucose, HbA1c, plasma lipids or body weight in diabetic db/db mice or sand rats. *In vitro* biological, biochemical and biophysical data suggest that the protein was correctly folded and biologically active.

**Conclusions/Interpretation:**

Recombinant adiponectin is ineffective at lowering blood glucose in diabetic db/db mice or sand rats.

## Introduction

Adiponectin is a 30 kDa protein (244–247 amino acids, dependent on species) secreted exclusively by adipose tissue [1]. It is homologous to complement factor C1q and contains a C-terminal globular head and a N-terminal collagen-like domain [1]. At least 3 different oligomeric adiponectin forms can be found in plasma: trimers, hexamers and high molecular weight (HMW) adiponectin [2]–[4]. Hexamers consist of two trimers that are linked together by a single disulfide bridge [3] whereas MHW adiponectin predominantly consists of 6 trimers [5]. The crystal structure of the trimeric globular form resembles the structure of TNFα [6] in spite of relatively low homology between the two proteins.

The plasma concentration of adiponectin is usually in the range of 1–20 mg/l which is high for a hormone. The adiponectin plasma concentration correlates inversely with diabetes, obesity and cardiovascular disease (CVD) [7]–[10]. In contrast, adiponectin levels seem to be increased in chronic inflammatory conditions where the adipose tissue mass is not increased, e.g., systemic lupus erythematosus, rheumatoid arthritis, inflammatory bowel disease, and cystic fibrosis [11].

Although a low (or high, in the case chronic inflammatory conditions) plasma adiponectin concentration appears to be a good marker for a number of pathophysiological conditions, the precise function and mechanism of action of adiponectin and its different oligomeric forms has proved elusive. Leptin deficient ob/ob mice with transgenic over expression of adiponectin in adipose tissue have normal levels of plasma glucose, insulin, non-esterified fatty acids and triglycerides even though the animals are significantly more obese than their non-transgenic ob/ob littermates [12]. Several groups have reported blood glucose lowering and/or insulin-sensitizing effects of recombinant full-length or globular adiponectin. The anti-hyperglycemic effect varies from study to study probably due to the fact that each study utilizes a unique combination of animal model (ob/ob, KKAy, FVB, C57BL/6J, Streptozotocin treated and high fat fed mice), adiponectin form (full-length and globular from mouse or man), expression host (*E.coli*, mammalian cells, *P. pastoris*) and dosing regimen [3], [13]–[19]. The reported effects of adiponectin injections or transgenic over-expression on body weight in mice are also variable, and anything from weight loss to weight gain has been reported [12], [14], [19]–[22]. Furthermore, over expression of adiponectin has been shown to reduce atherosclerosis in Apolipoprotein E deficient mice [19], [23].

On a cellular level it has been demonstrated that adiponectin potentiates the inhibitory effect of insulin on glucose production from primary hepatocytes [13]. Moreover, adiponectin has been reported to stimulate glucose uptake in muscle and fat cells as well as β-oxidation in muscle cells in an AMP kinase dependent manner [24]–[26]. Other cellular actions of adiponectin include anti-inflammatory effects on macrophages/monocytic cells [27]–[29] and endothelial cells [30], [31].

The purpose of the current study was to develop an effective expression and purification method for recombinant adiponectin and to evaluate the anti-diabetic potential of the different adiponectin forms in animal models of type 2 diabetes.

**Figure 1:**
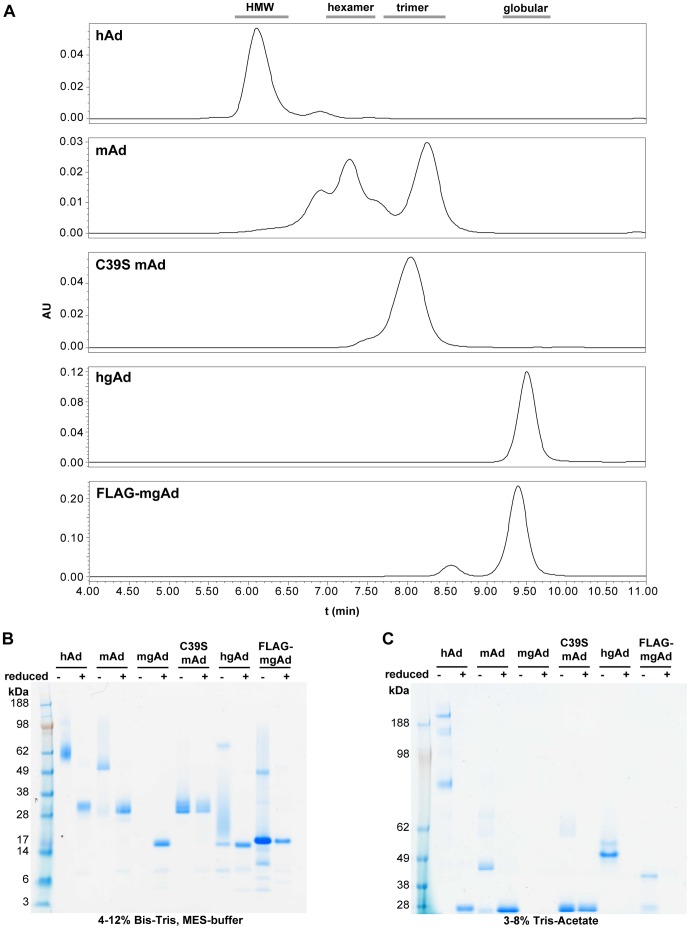
Characterization of adiponectin batches used for *in vivo* studies. **A.** Analytical size-exclusion chromatography of the batches. The grey bars at the top indicate the approximate elution positions of the various oligomeric forms of adiponectin. **B and C.** Coomassie stained SDS-PAGE analysis of the batches comparing reduced and non-reduced samples using a Bis-Tris (B) and a Tris-acetate (C) buffer system, respectively. Due to shortage of original sample mgAd was only analyzed reduced and on the Bis-Tris gel.

**Table 1:** Overview of the various forms of Adiponectin used in the described *in vivo* studies.

variant	species	fragment	mutation	expression host	form	R_h,av_ (nm)	M_w,av_ (kDa)	trimer/hexamer/HMW(%)	detected proline hydroxyl-ations	detected lysine hydroxyl-ations	total mass (Da)
hAd	human	full-length	–	CHOK1SV	HMW	10.3±0.1	447.5±0.5	0/2/98	71, 76, 95	33, 65, 68, 77, 101	mixture
mAd	murine	full-length	–	HEK293	trimer,hexamer	5.9±0.0	118.8±0.2	46/53/1	65, 79	36, 68, 71, 80, 104	25107
C39S mAd	murine	full-length	C39S	HEK293	trimer	5.3±0.0	75.5±0.1	100/0/0	47, 50, 56, 65, 79	68, 71, 80, 104	25975
mgAd	murine	Globular	–	HEK293	globular	3.0±0.1	47.5±0.1	100/0/0		104	16481
hgAd	human	Globular	–	*P. pastoris*	globular	–	–				16689
FLAG-mgAd	murine	Globular	V113M, N233Q	*S. cerevisiae*	globular	3.4±0.0	55.7±0.1	85/15/0			17054

The average hydrodynamic radius (R_h,av_) and average molecular mass (M_w,av_) are based on data from Multi-Angle Light Scattering experiments. Detected proline/lysine hydroxylations indicate amino acid positions where modifications were detected.

## Methods

### Cloning

The coding region of human adiponectin was amplified from Human Adipocyte Marathon Ready cDNA from Clontech (Mountain View, CA, USA) using the following primers: 5′- cgaagctttgccaccATGCTGTTGCTG-3 and 5′-ggaattCTCAGTTGGTGTCATGGTAG-3′, cloned into the pEE14.4 mammalian expression vector and transfected into CHOK1SV cells (Lonza Biologics, Basel, Switzerland).

**Figure 2:**
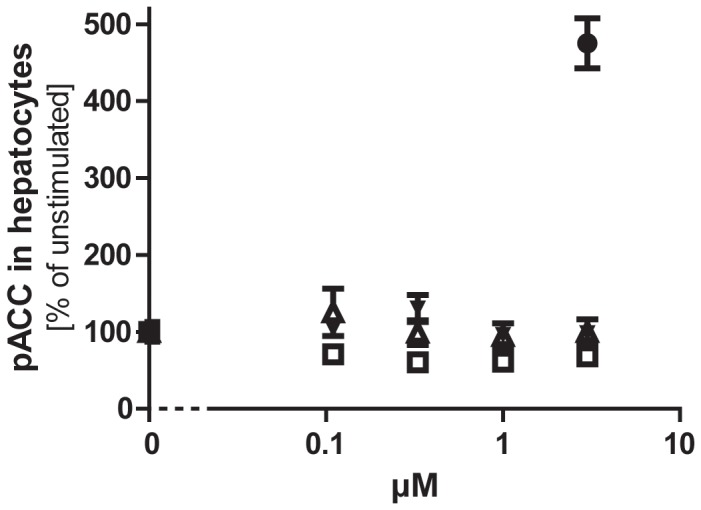
Stimulation of ACC phosphorylation in hepatocytes. Purified trimeric hAd (open squares), hexameric hAd (closed triangles), HMW hAd (open triangles) and 1 mM AICAR (closed circle, located at 3 µM) was used to stimulate ACC phosphorylation (pACC) in primary rat hepatocytes.

**Figure 3:**
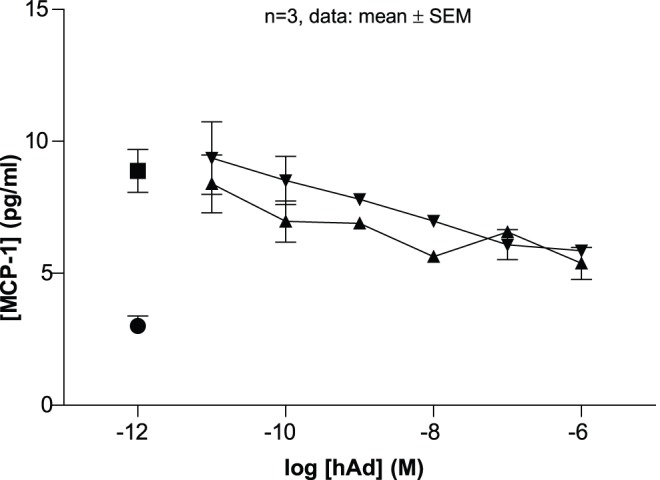
Inhibition of palmitate-induced MCP-1 production by THP-1 cells. hAd consistently and dose dependently inhibits the MCP-1 release. THP-1 cells were pre-incubated with 10 pM to 1 µM hAd for 2 h and subsequently stimulated with 100 µM palmitate for 24 h. Two different batches of hAd were compared (upward- and downward-pointing triangles) with controls with (square) and without (circle) palmitate.

The coding region of murine adiponectin was amplified from a Quick-Clone cDNA mouse spleen library from Clontech using the following primers: 5′-catcatcatgccgaagatgacgttactacaactgaag-3′, 5′-tcagttggtatcatggtagagaagaaagccagtaaatg-3′, cloned into the pJSV002 expression vector and transfected into HEK293-6E cells (Invitrogen, Carlsbad, CA, USA).

**Figure 4:**
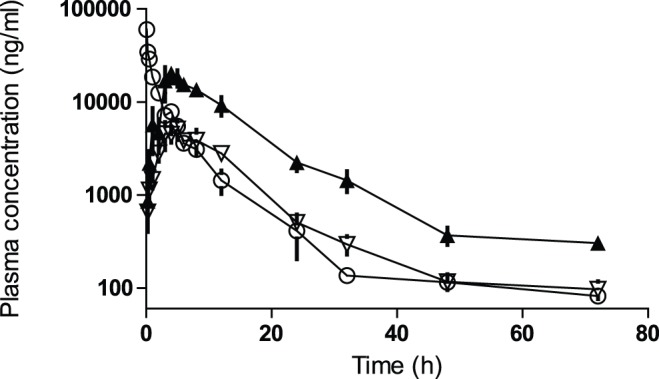
Pharmacokinetics of hAd in Sprague Dawley rats. At t = 0 hAd was dosed i.v. at 4.1 mg/kg (open circles), i.p. at 4.1 mg/kg (open triangles) or i.p. at 13.4 mg/kg (closed triangles). n = 2–5.

QuikChange site directed mutagenesis (Agilent Technologies, Santa Clara, CA, USA) was used to generate the mouse C39S version (primers: 5′-ggtccctccacccaagggaacttcagcaggttggatggcagg-3′, and 5′-cctgccatccaacctgctgaagttcccttgggtggagggacc-3′).

The original signal peptides were replaced by the human CD33 signal peptide.

**Figure 5:**
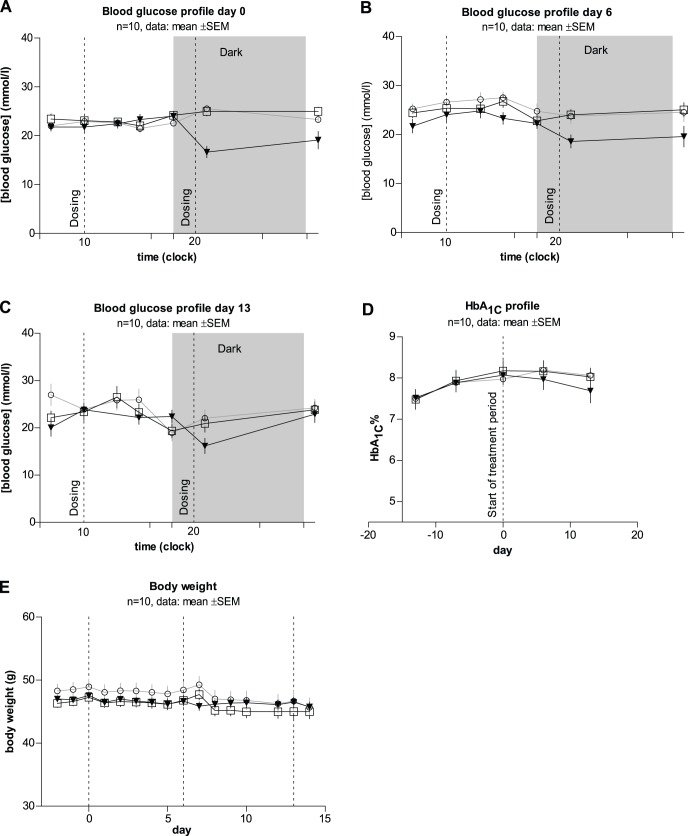
*In vivo* effects of hAd on glycaemic control in diabetic db/db mice. The animals were dosed intraperitoneally once daily with metformin (closed triangles) or twice daily with hAd (open squares) or vehicle (open circles) for 14 days. Blood glucose profiles were taken at day 0 (**A**), day 6 (**B**) and day 13 (**C**) of the study at 7 a.m., 10 a.m., 1 p.m., 3 p.m., 6 p.m., and 9 p.m. HbA_1c_ (**D**) was measured on the same days and additionally 7 and 14 days before the start of dosing. Body weight (**E**) was monitored daily. The data is representative for all studies performed with db/db mice. The data for all other forms of adiponectin is summarized in Table 2.

### Expression

Stable cell lines were selected using methionine sulfoximine (MSX, Lonza Biologics). A cell line expressing high levels of high-molecular weight adiponectin was chosen for protein production. HEK293-6E cells expressing murine adiponectin were cultured in FreeStyleTM medium (Gibco) for 5 days. Culture supernatants were harvested for protein purification.

**Table 2:** Data from db/db mice studies (mean ± SEM).

treatment	dose (mg/kg)	group size (n)	[HbA1c]day 0 (%)	Δ [HbA1c](%)	[blood glucose] day 0 (mM)	[bloodglucose] day 6 (mM)	[blood glucose]day 13 (mM)	body weight day 0 (g)	Δ body weight(g)	[TG]day 13 (mM)	[FFA] day 13 (µM)
**Study 1**											
vehicle	− b.i.d.	12	7.7±0.2	0.1±0.1	24.6±0.6	27.7±1.0	28.3±0.8	47.6±0.6	−2.3±0.5	2.4±0.2	295±29
C39S mAd	30b.i.d.	12	8.1±0.2	−0.1±0.1	24.3±0.8	28.4±0.9	27.7±1.0	46.6±0.6	−2.2±0.4	2.3±0.1	311±26
Metformin	400q.d.	12	8.3±0.2	−0.8±0.2 ***	24.6±0.7	23.6±2.5	23.3±1.3*	43.4±1.0**	−0.7±1.1	2.4±0.2	209±13
p			<0.14	<0.0009	<0.934	<0.075	<0.017	<0.0013	<0.17	<0.77	<0.026
**Study 2**											
vehicle	− b.i.d.	10	8.0±0.2	0.1±0.2	22.0±0.8	25.2±1.0	28.9±1.3	49.0±1.2	−1.9±0.6	1.9±0.1	237±23
hAd	30b.i.d.	10	8.2±0.3	−0.2±0.1	23.4±1.0	24.4±1.0	22.2±1.4	47.3±0.7	−1.7±0.6	1.8±0.1	217±14
mAd	30b.i.d.	10	7.8±0.2	0.1±0.1	22.6±0.7	24.1±1.2	23.1±1.0	46.2±1.2	−1.9±0.5	1.8±0.1	232±18
Metformin	400q.d.	10	8.1±0.2	−0.4±0.1*	21.8±0.8	21.7±1.4	20.1±1.9**	47.6±0.5	−0.6±0.4	2.0±0.1	234±20
p			<0.62	<0.029	<0.90	<0.18	<0.020	<0.13	<0.30	<0.86	<0.51
**Study 3**											
vehicle	− b.i.d.	10	7.1±0.2	0.1±0.1	23.1±1.1	27.0±1.3	23.0±1.5	47.3±0.7	0.6±0.4	2.1±0.2	346±21
mgAd	10b.i.d.	10	7.1±0.3	0.1±0.1	23.1±0.5	24.8±0.4	24.5±1.5	47.3±0.8	1.2±0.2	2.2±0.2	261±16
hgAd	10b.i.d.	10	7.1±0.2	0.1±0.1	23.1±1.0	26.1±1.3	24.9±1.2	47.8±0.8	0.9±0.4	1.8±0.1	326±37
FLAG-mgAd	15b.i.d.	10	6.8±0.3	0.1±0.1	22.1±0.8	23.6±1.1	24.0±0.9	47.4±0.9	1.2±0.4	2.0±0.1	337±46
p			<0.79	<0.99	<0.79	<0.15	<0.74	<0.90	<0.58	<0.18	<0.29

All adiponectin batches as well as the vehicle were dosed twice daily (b.i.d.), metformin was dosed once daily (q.d.) in the evening. All blood glucose measurements were performed in the morning (7 a.m.). Changes in blood glucose concentration and body weight (**Δ**) are given as the difference between day 0 and day 13.

**Table 3:** Data from diabetic *Psammomys obesus* study (mean ± SEM).

treatment	dose (mg/kg)	group size(n)	[HbA1c]day 0(%)	Δ [HbA1c](%)	[blood glucose] day 0(mM)	[bloodglucose]day 12 (mM)	body weight day 0(g)	Δ body weight(g)
vehicle	− (b.i.d.)	11	7.1±0.2	0.7±0.1	22.9±2.6	23.5±2.2	252.7±5.7	−6.3±4.2
mgAd	5 (b.i.d.)	11	6.5±0.3	0.7±0.1	20.2±2.4	21.6±2.9	243.9±4.4	0.5±1.8
hAd	15 (b.i.d.)	12	6.4±0.2	0.6±0.1	22.1±1.7	19.1±2.9	240.8±6.0	−0.2±2.0
p			0.16	0.58	0.70	0.54	0.30	0.20

All adiponectin batches as well as the vehicle were dosed twice daily (b.i.d.). All blood glucose measurements were performed in the morning (8 a.m.). Changes in blood glucose concentration and body weight (Δ) are given as the difference between day 0 and day 12.

Mouse globular adiponectin with an amino terminal FlagTag, DYKDDDDK (FLAG-mgAd) was cloned into the POT plasmid and expressed in the *Saccharomyces cerevisiae* strain ME1719 as previously described [32]. The coding sequence, corresponding to amino acids 109–247 of accession number Q60994 (SWISS-Prot Database), was synthesized by GENEART GmbH (Regensburg, Germany). An asparagine to glutamine substitution was introduced in position 233 to prevent N-glycosylation.

### Purification

The culture supernatant from the CHOK1SV cells was diluted with 1 mM CaCl_2_ in buffer A (20 mM Tris-HCl, pH 7.5). The protein was captured on a Q Sepharose FF column (GE Healthcare, Uppsala, Sweden) and eluted with 400 mM NaCl, 1 mM CaCl_2_ in buffer A. Peak fractions were pooled and further purified on a Source 15Q (GE Healthcare) column using a gradient of NaCl in 1 mM CaCl_2_/buffer A. The HMW form of adiponectin was isolated on a Superdex 200 HR 26/60 using 5 mM CaCl_2_, 150 mM NaCl in buffer A as eluent.

The culture supernatant of HEK293-6E cells expressing murine adiponectin was adjusted to 1 M ammonium sulphate in buffer A. The protein was captured on a Phenyl Sepharose Fast Flow column (GE Healthcare) and eluted with buffer A. Peak fractions were pooled and diluted 5-fold with buffer A. The diluted pool was further purified on a Q Sepharose HP (GE Healthcare) column using a gradient of NaCl in buffer A. The wildtype protein was further purified on an SP Sepharose HP column (GE Healthcare) using a gradient of NaCl in 10 mM citric acid pH 4.0.

The supernatant of *S. cerevisiae* expressing FLAG-mgAd was adjusted to pH 7.5 and applied to a Q Sepharose FF column and eluted with a gradient of NaCl in 1 mM CaCl_2_/buffer A. Peak fractions were pooled and further purified by Superdex 200 gelfiltration.

*P. pastoris* supernatant containing hgAd was desalted, adjusted to 1 M ammonium sulphate in buffer A, loaded onto a Butyl Sepharose FF column (GE Healthcare) and eluted with buffer A. The pool was desalted against the same buffer and further purified on a Source 30Q column (GE Healthcare). The product was eluted by a gradient of NaCl in buffer A, concentrated by ultrafiltration and further purified by Superdex 200 gelfiltration. Alternatively the protein was purified as previously described Liu et al. 2007] followed by a gelfiltration with a Superdex 75 HR 26/60 column (GE Healthcare) using 5 mM CaCl_2_, 150 mM NaCl in buffer A as eluent.

### Endotoxin Assay

All preparations of adiponectin were tested for endotoxins by a kinetic turbidometric assay using Limulus Amebocyte Lysate (Charles River Laboratories, Wilmington, MA, USA) and contained less than 5 endotoxin units/mg.

### Analytical Size-exclusion Chromatography

Analytical SEC was carried out using a 300×7.8 mm BioSep-SEC-S3000 column (Phenomenex, Torrance, CA, USA). The column was equilibrated in 0.2 M Tris-HCl pH 7.8. Proteins were eluted using isocratic elution at a flow rate of 1 ml/min. Eluted proteins were detected by UV absorption at 280 nm. A standard Waters Alliance 2695 separation unit equipped with a Waters 2487 dual UV absorbance detector (Waters, Milford, USA) was used for all separations.

### Dynamic Light Scattering

Samples (4 mg/ml) were centrifuged at 13,000×g for 10 minutes and 25 µl dispensed to a 384 well plate. The plate was centrifuged at 1000×g and analysed in a DynaPro™ Plate Reader Plus (Wyatt Technology Corporation, Santa Barbara, CA, USA). Each well was measured 20 times with 10 second acquisitions at 25°C. The hydrodynamic radius (R_h_) of the sample was obtained from the measured translational diffusion coefficient using the Stokes-Einstein relation from culumants analysis and presented as R_h,average_. Analysis was performed by DYNAMICS software from Wyatt Technology Corporation.

### AF4-MALS

Asymmetric-Flow Field-Flow Fractionation Multi-Angle Light Scattering (AF4-MALS) was performed using an Agilent 1200 liquid chromatography system (Agilent Technologies, Palo Alto, CA). In addition the system was equipped with an Eclipse 3 Separation System, a DAWN HELEOS MALS detector and an Optilab rEX refractive index detector (Wyatt Technology Corporation). A Short Channel with a spacer height (wide) of 350 µm with polyethersulfone (PES) membranes with a cut-off of 10 kDa was used. The channel flow rate was set to 1 ml/min and the cross flow rate was constant at 1.3 or 2 ml/min. 20 mM Tris-HCl pH 7.4 including 100 mM NaCl and 1 mM CaCl_2_ was used as buffer. Weight average molar mass (M_W_) was determined by using the software ASTRA® V (Wyatt Technology) using a refractive index increment (*dn/dc*) of 0.185 mL g^−1^.

### 1D-SDS-PAGE

Samples for 1D-SDS-PAGE were diluted and mixed with SDS-sample buffer without heating. 4–12% Bis-Tris gel with MES-running buffer and a 3–7% Tris-Acetate with Tris-Acetate running buffer (Novex, Invitrogen, Carlsbad, USA) were used for separation. After electrophoresis the gels were stained with Instant Blue (Expedeon, Harston, UK) according to the manufacturer instructions.

### AMPK Activity in Primary Hepatocytes

Hepatocytes were isolated from anaesthetized male Sprague Dawley rats using a two-step collagenase perfusion technique. The cells were plated onto collagen-coated plates in basal medium (Medium 199, 5.5 mM glucose, 0.1 mM dexamethasone, 100 units/ml penicillin, 100 mg/ml streptomycin, 2 mM L-glutamine ) with the addition of 1 nM insulin and 4% FCS. The medium was replaced with basal medium containing 1 nM insulin and 4% FCS 1–2 hours after initial plating in order to remove dead cells. Cells were stimulated the following day in basal medium with or without adiponectin for 60 minutes and lysed by adding ice-cold cell extraction buffer (Biosource) and incubating 30 min on ice. AMPK activity was measured as phoshorylated ACC by ELISA. In brief lysate was added to streptavidincoated 96 well plates (Pierce) and incubated o/n at 4C. Plates were washed (TBS-T), anti-pS79ACC 1∶3000 in TBS (Upstate ) was added and the plates incubated for 3 hours at rt. The plates were then washed (TBS-T), anti-rabbit-IgG-HRP 1∶3000 in TBS (Immunopure cat# 31460) was supplemented and incubated 1 hour at rt. HRP activity was visualised using chromogen and stop buffer from Biosource followed by detection via ELISA reader.

### MCP-1 Release from THP-1 Cells

THP-1 cells (ATCC) were cultured as described by the supplier. Cells were plated in 96 well plates (50,000 THP-1 cells/well) and stimulated with adiponectin (10 pM–1 µM) for two hours prior to 24 h palmitate (100 µM) challenge. Media was isolated and human MCP-1 was detected using a LincoPlex kit (Millipore, Billerica, MA).

### Animals

Male db/db mice (Taconic Europe) weighing from 45 to 50 g were randomized according to non-fasted blood glucose levels,glycated hemoglobin (HbA1c), and body weight. Mice were were fed ad libitum (Altromin 1324 standard diet, Brogaarden, Denmark) and dosed from weeks 11–12.

Male sand rats (Psammomys obesus) from Harlan Laboratories, Jerusalem, Israel had ad libitum access to low energy diet (3084–111507, 2.5 kcal/g, Harlan Teklad). They were fed high energy diet (HE diet, Formulab Diet 5008, 3.5 kcal/g, Lab Diet) for a test-period of 10 days. Animals that responded to the HE diet with mean blood glucose >10 mM were fed HE diet for additional 3 weeks to induce stable diabetes. The diabetic animals were randomly assigned to three treatment groups.

All animals were housed at 23°C under standard conditions in a 12∶12 h light/darkness cycle. Principles of laboratory animal care were followed and study approval was obtained from the Animal Experiments Inspectorate, Danish Ministry of Justice. (Dyreforsøgstilsynet, Slotsholmsgade 10, 1216 København K, Denmark).

### *In vivo* Studies

The db/db mice were dosed intraperitoneally (ip) once (metformin) or twice (adiponectin or vehicle) daily at 10 a.m. and 8 p.m. for 14 days. HbA1c was measured on day 0, 6 and 13. Blood glucose was measured at 7 a.m., 10 a.m., 1 p.m., 3 p.m., 6 p.m., and 9 p.m. on day 0, 6 and 13. Plasma concentrations of free fatty acids and triglycerides were determined at the end of the study.

Nineteen weeks old diabetic Sand rats were dosed ip twice daily and received either vehicle, mgAd, or hAd, respectively at 8 a.m. and 6 p.m. All sand rats treated with adiponectin were kept on HE diet throughout the study. Body weight and blood glucose, taken in the unfasted state before the morning dose, were measured before the start of the study and four times during the dosing period. HbA1c was measured before the start and at the end of the study period.

Pharmacokinectis was studied in male Sprague Dawley rats (∼450 g). Administration was done either ip or iv at t = 0 and blood samples were taken at t = 0.083; 0.25; 0.5; 1; 2; 3; 4; 5; 6; 8; 12; 24; 32; 48; 72 h using a sparse sampling protocol.

### Analysis of Samples from *in vivo* Studies

Blood glucose was measured in 10 µl full blood samples taken from the tip of the tail by puncturing the capillary bed with a lancet, using a 10 µl heparinised capillary tube to sample the blood. The capillary tube was then shaken into 500 µl glucose/lactate System Solution and measured in a Biosen, autoanalyser (EKF Diagnostics GmbH, Magdeburg, Germany) according to the manufacturer’s instructions.

HbA1c was measured in 5 µl full blood sample taken from the tip of the tail by puncturing the capillary bed with a lancet, using a heparinised capillary tube to sample the blood. The capillary tube was then shaken into 500 µl Hitachi Hemolyzing Reagent and measured in a Hitachi 912 autoanalyser (Roche A/S Diagnostics, Mannheim, Germany), according to the manufacturers instructions.

Plasma triglycerides and free fatty acids were also measured using Hitachi 912 autoanalyser according to manufactures instructions.

### Measurement of Recombinant Adiponectin in Mouse Plasma

Human adiponectin in mouse plasma was measured by ELISA. Taking advantage of the multimeric nature of adiponectin the same monoclonal antibody MAB1065 (R&D systems) was used as both catcher and detector. MAB1065 is directed against the globular part of human adiponectin and does not cross-react with the murine counterpart.

## Results

### Generation and Characterization of Recombinant Adiponectin Constructs

The recombinant adiponectin batches generated for this study include the carboxy-terminal globular domain, low-molecular weight (LMW) as well as high molecular (HMW) forms. Murine and human adiponectin forms were expressed in a variety of microbial and mammalian host systems. Table 1 provides an overview of the constructs that were tested *in vivo.*

All batches underwent extensive biochemical and biophysical characterization to ensure both purity and integrity of the samples after the purification procedure, but also to verify that the proteins behaved as expected and as previously described for similar constructs. The experimentally determined properties of the various forms are summarized in Figure 1 and Table 1.

Full length human adiponectin (hAd) was expressed especially well in mammalian CHOK1SV cells, and an expression level of several grams per litre of conditioned media was obtained. Purification yielded a homogeneous preparation of HMW adiponectin. Asymmetric-Flow Field-Flow Fractionation Multi-Angle Light Scattering (AF4-MALS) measurements showed a molecular mass of 448 kDa, which is in good correspondence with an 18-mer. Moreover, we could detect the previously described hydroxyprolyl and glycosylated hydroxylysyl modifications of the collageneous domain in our preparation.

Full length murine adiponectin (mAd) was produced transiently in HEK293T cells. This expression system predominantly gave trimeric and hexameric mAd. As no size fractionation was applied, this mixture of oligomeric forms was preserved throughout purification. The C39S version of the same construct (C39S mAd) was expressed exclusively as a trimer.

Murine globular adiponectin (mgAd) was either produced by trypsin cleavage of mAd or C39S mAd, or expressed with an amino-terminal FLAG-tag in *Saccharomyces cerevisiae* (FLAG-mgAd, with a mutation of asparagine 233 to glutamine to prevent unwanted N-glycosylation). Moreover, we generated tagless human globular adiponectin (hgAd) by expression in *Pichia pastoris*, as previously described [17].

It should be noted that all of our preparations have been monitored for endotoxin content. The majority of batches had endotoxin levels well below 1 EU/mg and no batch was above 5 EU/mg.

### *In vitro* Biological Evaluation

Full-length adiponectin has previously been shown to activate AMP kinase in hepatocytes *in vitro* [33]. To evaluate the biological activity of our recombinant adiponectin batches, we accordingly decided to test the effect on AMP kinase activity in primary rat hepatocytes. Surprisingly, none of our batches led to an increase in phosphorylated ACC in hepatocytes *in vitro* (data shown for purified HMW, trimeric and hexameric hAd in figure 2). It should be mentioned that some low purity adiponectin batches caused an increase in phosphorylated ACC in hepatocytes *in vitro*. However, this effect turned out to be due to contamination e.g. with glycerol from certain spin filters (data not shown). Since our recombinant adiponectin batches previously have been shown to possess immuno-modulatory properties [27] we decided to test the effect of hAd on LPS- and palmitate stimulated production of MCP-1 in THP-1 cells. Figure 3 shows a dose dependent inhibition of MCP-1 production by two different batches of hAd.

### *In vivo* Evaluation in db/db and ob/ob Mice

The pharmacokinetics properties of hAd was studied in detail in Sprague Dawley rats (figure 4). The half-life of hAd was approximately 6 hours and the maximum plasma concentration (approximately 20 mg/l in the 13.4 mg/kg group) was reached after 5–6 hours. Plasma exposure in db/db mice was assessed by single time point measurements due to the limited amount of blood that can be drawn from a mouse. Plasma exposure 6 hours after a single i.p. injection of 30 mg/kg hAd was approximately 100 mg/l and the plasma exposure of hgAd in db/db mice 3 hours after a single i.p. injection of 10 mg/kg hgAd was approximately 70 mg/l. The antibody employed for the measurement of hAd and hgAd was specific for human adiponectin and did not cross-react with endogenous rat or mouse adiponectin.

Initially, we tested the acute effect of full-length adiponectin produced in *E. coli* and mammalian cells (CHO and HEK293 cells) on blood glucose in diabetic ob/ob and db/db mice. Briefly, two batches of *E. coli* produced adiponectin and four batches of mammalian cell produced adiponectin were injected i.p. at 30–40 mg/kg to diabetic mice (n = 9–13 per group). However, none of the six batches were capable of lowering blood glucose (data not shown). The adiponectin doses chosen for this experiment as well as the experiments mentioned below were based on the highest doses employed in previously published studies (13;17).

The failure of the six adiponectin batches to reduce blood glucose acutely in diabetic ob/ob and db/db mice prompted us to conduct sub-chronic experiments in diabetic db/db mice. The data from one of these experiments are shown in Figure 5 as an example. As can be seen, hAd injected i.p. b.i.d. was unable to reduce blood glucose on day 0, 6 and 13. Moreover, hAd did not affect HbA1c or body weight. Metformin (positive control) was injected i.p. q.d. at 8 p.m. and lowered blood glucose significantly at day 0, 6 and 13. The lack of blood glucose measurements between 9 p.m. and 6 a.m. the following morning leads to an underestimation of the blood glucose lowering effect of metformin.

The data from sub-chronic testing in diabetic db/db mice are summarized in Table 2. As can be seen from the table neither C39S mAd, hAd, mAd, muse globular adiponectin (mgAd), human globular adiponectin (hgAd), nor FLAG tagged mouse globular adiponectin (FLAG-mgAd) had any effect on HbA1c, blood glucose on day 0, 6 and 13, body weight, plasma triglycerides (TG) or plasma free fatty acids (FFA) after 2 weeks of i.p. dosing.

### *In vivo* Evaluation in Sand Rats

The hyperphagic and hyperglycaemic characteristics of both the ob/ob and db/db the mouse models are caused by a deficient leptin system (i.e., a lack of leptin or the leptin receptor). To test whether the lack of a blood glucose lowering effect by adiponectin was specific for db/db (and ob/ob) mice and potentially dependent on a normal regulation of leptin, we decided to test two adiponectin batches in diabetic *Psammomys obesus* (sand rats). As shown in Table 3, neither mouse globular adiponectin (mgAd) nor wild-type human adiponectin (hAd) had any effect on HbA1c, blood glucose on day 0 and 12 or body weight after 13 days of i.p. dosing.

## Discussion

In the present study, we show that high expression levels (>1 g/l) of secreted soluble full-length adiponectin can be obtained, when CHOK1SV cells are used as an expression host. The CHOK1SV cells are capable of producing all three adiponectin forms (trimeric, hexameric and HMW adiponectin), but the relative production of each form varies from clone to clone. Transiently transfected HEK293 cells predominantly produce trimeric and hexameric adiponectin. Our extensive biochemical and biophysical characterization has revealed that the batches used in this study were homogeneous, of the expected size - both in terms of the single amino acid chains and the degree of oligomerization, and contained the previously described post-translational modifications.

We also show that CHO cell-produced human adiponectin (hAd) is functional *in vitro* as it dose-dependently inhibits palmitate stimulated MCP-1 release in THP1 cells. Moreover, our hAd has previously been shown to promote an anti-inflammatory phenotype in human and mouse macrophages [27]. Furthermore, it should be mentioned that another lab recently has observed convincing immuno-modulatory properties of our recombinant adiponectin (Parth Narendran, personal communication). Taken together, these findings suggest that the described *in vivo* studies were carried out with recombinant adiponectin that was biochemically intact, correctly folded, and biologically active. Even though the literature suggests that full-length adiponectin can stimulate the AMP kinase activity in hepatocytes [33], we did not see any effect of our full-length batches. However, it is our experience that contaminating factors (e.g. glycerol) in protein preparations easily can increase the level of pACC in cells, which can make AMP kinase activity a slightly tricky endpoint for the assessment of cellular effects.

Collectively, we have conducted six acute experiments in diabetic db/db or ob/ob mice and six two-week subchronic experiments in diabetic db/db mice. The animals were dosed with mouse or human full-length or globular adiponectin batches. A proportion of full-length batches consisted primarily of the trimeric and/or hexameric form (mAd and C39S mAd) whereas other batches consisted mainly of HMW adiponectin (hAd). Plasma exposure of human full-length adiponectin reached 5–10 times the level reported for healthy mice. However, we failed to detect any blood glucose lowering effect in any of the experiments. This observation leads us to surmise that exogenously administered recombinant adiponectin is ineffective in lowering plasma glucose in leptin deficient mouse models of type 2 diabetes. This conclusion is supported in part by a recent study where adenovirus mediated overexpression of adiponectin was relatively ineffective in improving glucose clearance in ob/ob mice in spite of a three-fold increase in the plasma adiponectin level [34]. In contrast, two earlier studies have shown an anti-diabetic effect of adiponectin in ob/ob mice. In the first of these full-length mouse adiponectin produced in HEK293 cells lowered blood glucose acutely [13]. However, only four animals per treatment group were used in that study. From our experience, it is difficult to achieve a definitive conclusion in this model when such a small number of mice per group is employed. In the second ob/ob study, transgenic overexpression of adiponectin in adipose tissue prevented development of diabetes and hyperlipidemia [12]. However, this was a prevention study where animals were exposed to a high adiponectin level from birth while our findings are based on treatment of animals with established diabetes. Accordingly, the conclusion drawn from the latter study does not necessarily conflict with our findings. It is also possible that adiponectin is required to be processed by adipocytes, in a currently unknown manner, in order for it to be metabolically active.

The blood glucose lowering effects of adiponectin reported elsewhere show some variability. This might be attributable to the fact that each study utilizes a unique combination of animal model (KKAy, FVB, C57BL/6J, Streptozotycin treated and high fat fed mice), adiponectin form (Full-length and globular from mouse or man), expression host (*E.coli*, mammalian cells, *P. pastoris*) and dosing regimen [3], [14]–[19].

To test the anti-diabetic effect of adiponectin in a non-leptin deficient model, we decided to dose adiponectin i.p. b.i.d. to sand rats. Once more, we failed to detect any blood glucose lowering effect.

Thus, we conclude that our recombinant adiponectin preparations are ineffective in lowering blood glucose in animal models of type 2 diabetes. This conclusion is supported to some extent by the fact that numerous pharmaceutical and biotech companies (e.g. Protemix, Merck KGaA (Merck Serono), Maxygen) which have worked on adiponectin over the past decade, have been unable to progress their research projects beyond the pre-clinical stages.

Finally, a note of caution should be made with regard to endotoxin contamination of protein preparations that are injected in diabetic animals. Examination of the literature reveals that approximately half of the published studies where adiponectin was administered to diabetic rodents fail to mention whether or not endotoxin levels were monitored, or whether measures were taken to avoid or remove endotoxin contamination. At the same time, endotoxins have been reported to lower blood glucose in a number of mouse strains [35]. From our experience, endotoxins are almost invariably introduced upon handling and purification of proteins if no specific measures are taken to prevent it. This is also true for samples from endotoxin-free expression systems such as mammalian cells and yeast. Common sources include bacterial contaminations in columns, containers and purification equipment. It is crucial to use disposable material wherever possible and sanitize all other equipment thoroughly, e.g., by use of concentrated base. It should also be noted, that we have not been able to remove existing endotoxin contaminations from preparations of adiponectin expressed in mammalian systems, possibly due to affinity of lipopolysaccharaides to the glycosylations. Accordingly, researchers in diabetes should be encouraged to report endotoxin levels when they purify proteins for the purpose of injection into animals. In this instance, it should be emphasized that all adiponectin batches employed in the current study had very low levels of endotoxins.

In conclusion, our work suggests that adiponectin is ineffective with regard to lowering blood glucose in mice and Sand rats. This raises the question: What is the primary biological function of adiponectin? So far the data from testing of our adiponectin batches by other labs has generated support in favour of the conclusion that adiponectin is an immunomodulatory molecule [27] (Parth Narendran, personal communication).
